# Supplementary material for: Pre-collecting lymphatic vessels form detours following obstruction of lymphatic flow and function as collecting lymphatic vessels
Source: PLoS One. 2020 Jan 15;15(1):e0227814. doi: 10.1371/journal.pone.0227814 (PMC6961945; doi:10.1371/journal.pone.0227814)
Supplement: Attachment — Submitted filename: Response_to_Reviewer_2019.11.4.docx [file pone.0227814.s001.docx]

Dear Editor:

Thank you and the reviewers for these important comments and advice concerning this manuscript. We have revised our paper according to the reviewers’ comments.

We have revised the following sections:

**Discussion**: We have described the formation and characteristics of the lymphatic detour from the present results, we have added references, and we discuss our findings that show the detour is useful to decrease lymphedema.

**Introduction:** As the discussion has been revised, we have also revised the Introduction section accordingly and have add animal and human references.

We have got English proofreading in our revised manuscript, and revised the following sections:

**Title of this manuscript:** We have changed “Connecting lymphatic vessels form detours upon obstruction of lymphatic flow and function as collecting lymphatic vessels.” to “Connecting lymphatic vessels form detours following obstruction of lymphatic flow and function as collecting lymphatic vessels.”

**Acknowledgements:** We have added “We would like to thank Editage (www.editage.com) for English language editing.” on page 27, lines 471-472 because the company requests us.

We kindly request you to please review our newly revised manuscript.

**Reviewer #1**:

This is a very interesting manuscript about the formation of detours by connective lymphatic vessels after obstruction of lymphatic flow in mice. In addition, it is a current and important problem for patients suffering from lymphedema. The Abstract and Introduction are in line with the work presented and are clearly organized.

Q1. However, the authors should clarify in the Material and Methods section if the statistical analyzes were performed and how they were made. In addition, the authors do not mention these analyzes in the Results section. Therefore, the statistical analyzes details should be clarified to ensure that readers understand exactly what the researchers studied. Also, the authors should include more information that clarifies and justifies their choice of methods.

**Response 1.**

Thank you for your comments.

As you have mentioned, it is important to state how statistical analyses were performed in the Materials and Methods section. However, as Reviewer 2 has also judged, we consider that statistical analysis was not applicable in this study. We provide the following two reasons for not having undertaken statistical analyses in this study.

First, we do not compare any results from Tables 1 and 2 in the Results and Discussion sections. We only classified the patterns of lymphatic flow after lymphatic ligation, and we have focused mainly on detours after lymphatic ligation in the Results and Discussion sections.

Second, we were not able to perform statistical analysis concerning results shown in Tables 1 and 2 because the numbers of each pattern are too small (e.g. 0 or 1).

As such, we did not perform any statistical analysis in this study.

Additionally, we have revised the ICG lymphography results. We consider that the description is now easier to understand. We refer you to pages 13-14, lines 226-248.

Q2. In addition, the authors need to be bolder and more analytical in the Discussão section, using more area references to expand the discussion and make it even more interesting. Regarding the Conclusions section, conclusions must be drawn appropriately based on the data presented, not just a repetition of the data found.

**Response 2**.

We have revised the Introduction and Discussion sections in accordance with the reviewers’ comments, which we consider have now greatly improved our manuscript. Kindly refer to our newly revised changes.

Minor points:
1. Line 93, page 6. It is written "the cranial ligation site and the caudal ligation site." Were these points chosen in a specific or random way? It is not clear.

**Response 3.**

We have added a sentence on page 9, lines 141-142, and our changes are highlighted in red.

2. Line 127, page 8. "... with 1.75% isoflurane..." is written in different letters from the rest of the text. Please, check the text formatting.

**Response 4.**

We have now corrected this on page 11, line 180, and our changes are highlighted in red.

3. Line 148, page 9. It is written that "... stapled onto transparent plastic sheets...". Did the plastic allow the correct fixation of the collected material?

**Response 5.**

We have added a sentence on page 12, lines 201- 204, and our changes are highlighted in red.

4. Line 287, page 17. Reference 15 is not enclosed in square brackets as the rest of the references.

**Response 6.**

Number of Refence 15 was changed to Reference 32. We have enclosed reference 32 in square brackets on page 23, line 399, and our changes are highlighted in red.

5. Figure legends need to be better organized. For example, after (A) do not use the end point [(A) Evans blue dye ...]. Another example, do not use a semicolon (;) to separate the scale bar from its size [you can use Scale bar of 1 cm]. To indicate objects in the figure, do not separate their name from their function using a semicolon (;), you can use a colon (:) and to separate the different objects in the figure you use semicolons (;) [example: Open white arrowheads: incision site; asterisk: inguinal LN]. Please you should make these changes in all captions of the figures.

**Response 7.**

We have undertaken to make all of the recommended amendments to the figure legends, and our changes are highlighted in red.

**Reviewer #2**:

The authors observed detour of lymphatics (collateral formation?) in mice after lymphadenectomy in their previous study (ref 8). They aimed to investigate this detour phenomena in this current study by ligation of the lymphatic vessel in distal and proximal portions in 51 mice. The authors used ICG lymphography to image the detour formation and performed histological analysis of the detoured vessel. 5-ethynyl-2’-deoxyuridine (EdU) was used to evaluate any evidence of lymphangiogenesis. The authors found that detour was formed by 30 days after ligation and the detoured vessels were not formed by lymphangiogenesis. They concluded that the detour of lymphatic vessels might be useful for effective care of lymphedema.

Although investigation of structural changes of the lymphatic vessel after segmented obstruction with ligation in mice may be unique, there are several fundamental questions about this study.
Q1. In line 91, the authors described that they developed detours by excision of the inguinal lymph node but the pattern of detours by this surgery was unexpected in their previous study. However, they did not explain what was unexpected. This finding was the reason for current study design and therefore it requires further explanation.

**Response** **1**.

Thank you for your comments.

We have revised the sentence on pages 8-9, lines 135-139, and our changes are highlighted in red.

Q2. From line 66 to 72, patients with lymphedema demonstrated collateral formation and structural changes but the authors said detours of the lymphatics after the lymphadenectomy in mice suggested that these detours prevented lymphedema. There was contradiction between stories in patients and mice.

**Response 2.**

We have made significant changes to the Introduction, which is now ready for your further consideration. Pages 4-6, lines 53-82 correspond to almost all of your comments.

Q3. From line 82 to 87, 8 mice were used for lymphography and histological evaluation on day 30. 28 mice were divided into 7 groups (n-4 each) and used by the same evaluation at different time points (Day 10, 12, 15, 18, 21, 24, and 27). However, Results in Table 1 showed 8 mice studied at different time points (Day 15, 12, 27 and 30). Procedures in 8 mice were missing in the Materials and Methods section.

**Response 3.**

We have revised the animals in the Materials and Methods section, on page 8, lines 121-132, and our changes are highlighted in red. Kindly refer to our newly revised changes.

Q4. In line 10, lymphatic routes after the ligation were divided into three patterns; detours, original route and no detection. The detours were discussed in the Discussion but the other two patterns were not discussed. As for technical aspect, I think 6-0 nylon was too thick to ligate the lymphatic vessels in mice. I recommend using 9-0 or 10-0 for this purpose.

**Response 4.**

We have made many revisions to the Discussion section. Kindly refer to pages 21-23, lines 367-396, in response to your comments. In our next experiment, we intend to use a 9-0 or 10-0 suture.

Q5. In line 232, the authors concluded that these detours were not developed by lymphangiogenesis. This might be true for this study, however I don’t think EdU is adequate enough to appreciate lymphangiogenesis at this stage. There is a substantial body of research that highlights lymphangiogenesis after surgical procedures in different animals including mice, rats, rabbits, digs, pigs and sheep. It is insufficient to conclude the absence of lymphangiogenesis on the basis of negative EdU cells alone.

**Response 5.**

Thank you for your valuable advice. We greatly appreciate your comments, which we agree with. In future studies, we intend to use another method to detect lymphangiogenesis or dilatation of existing connecting lymphatics forming lymphatic detours.

Q6. In line 313, the authors described no obvious evidence of lymphatic reconstruction of the lymphatic route in humans. This statement is inaccurate. Alterations of the lymphatic pathways in patients with lymphedema have been examined with lymphangiography, lymphoscintigraphy, ICG lymphography, MR lymphography, and SPECT/CT. Each clinical imaging has specific criteria to diagnose lymphedema.

**Response 6.**

In response to your helpful comments, we have significantly revised the Discussion and Introduction sections. We have added a lymphatic image of human lymphedema to pages 4-5, lines 53-74 of the Introduction section, and we have revised part of the Discussion section on pages 25-26, lines 436-454 for your consideration.

Q8. Overall, the manuscript requires further explanation about background of the study design in Introduction and detailed procedures in Materials and Methods. A strong point of this study was combination of ICG lymphography and immune-histochemical analysis and weak points were clinical correlations and lack of functional testing.

**Response 8.**

We have considerably revised the Introduction and Discussion sections according to the reviewers’ comments. We consider that the reviewers’ comments have helped us to improve our manuscript.
